# *Mycobacterium tuberculosis* Rv3160c is a TetR-like transcriptional repressor that regulates expression of the putative oxygenase Rv3161c

**DOI:** 10.1038/s41598-021-81104-y

**Published:** 2021-01-15

**Authors:** Hasan Tükenmez, Souvik Sarkar, Saber Anoosheh, Anastasiia Kruchanova, Isabel Edström, Gregory A. Harrison, Christina L. Stallings, Fredrik Almqvist, Christer Larsson

**Affiliations:** 1 Department of Chemistry, Umeå University, 90187 Umeå, Sweden; 2 Department of Molecular Biology, Umeå University, 90187 Umeå, Sweden; 3 Umeå Centre for Microbial Research, Umeå University, 90187 Umeå, Sweden; 4 Molecular Infection Medicine, Sweden (MIMS), Umeå University, 90187 Umeå, Sweden; 5 Department of Molecular Microbiology, Washington University School of Medicine, St. Louis, MO 63110 USA; 6 Present Address: Holmsund, Sweden

**Keywords:** Tuberculosis, Target identification, Target validation, Gene regulation

## Abstract

Tuberculosis, caused by *Mycobacterium tuberculosis* (*Mtb*), is a major health threat listed among the top 10 causes of death worldwide. Treatment of multidrug-resistant *Mtb* requires use of additional second-line drugs that prolong the treatment process and result in higher death rates. Our team previously identified a 2-pyridone molecule (C10) that blocks tolerance to the first-line drug isoniazid at C10 concentrations that do not inhibit bacterial growth. Here, we discovered that the genes *rv3160c* and *rv3161c* are highly induced by C10, which led us to investigate them as potential targets. We show that Rv3160c acts as a TetR-like transcriptional repressor binding to a palindromic sequence located in the *rv3161c* promoter. We also demonstrate that C10 interacts with Rv3160c, inhibiting its binding to DNA. We deleted the *rv3161c* gene, coding for a putative oxygenase, to investigate its role in drug and stress sensitivity as well as C10 activity. This *Δrv3161c* strain was more tolerant to isoniazid and lysozyme than wild type *Mtb*. However, this tolerance could still be blocked by C10, suggesting that C10 functions independently of Rv3161c to influence isoniazid and lysozyme sensitivity.

## Introduction

Tuberculosis (TB) caused by *Mycobacterium tuberculosis* (*Mtb*) is still a major infectious disease worldwide^1^. In 2018, an estimated 10 million people fell ill with TB and 1.5 million died globally^1^. The fight against TB is exacerbated by the emergence of multidrug-resistant strains (MDR-TB) resistant to at least two of the first-line drugs to treat TB, isoniazid (INH) and rifampicin^1^. These infections require additional second-line drugs and longer duration of treatment where the treatment success rate drops substantially^1^. Over the last 50 years, only three new TB drugs have been developed and approved for use against TB: bedaquiline, delamanid and most recently, pretomanid. However, there are already several cases reported with TB resistant to both bedaquiline and delamanid^2–5^. Therefore, there is still a desperate need to develop new strategies to target drug-resistant *Mtb*.

Previously, our team described a thiazolo ring-fused 2-pyridone (C10) with the ability to increase sensitivity to oxidative stress, acid stress and the first-line antibiotic INH^6^. C10 was found to potentiate the bactericidal activity of INH and prevent the occurrence of INH-resistant mutants^6^. Moreover, INH-resistant *katG* mutants were re-sensitized to INH in the presence of C10, demonstrating that INH resistance can be circumvented^6^. We also showed that C10 inhibited respiration in *Mtb*, and previous reports have shown that both inhibition and stimulation of *Mtb* respiration can potentiate bactericidal activity of INH^7,8^. However, the link between C10 effects on respiration and C10 effects on INH sensitivity were not elucidated. The C10 target(s) in *Mtb* as well as molecular mechanism of the INH potentiation is unknown.

Here, we discovered the *rv3160c-rv3161c* operon to be induced by C10 in *Mtb*. We found that C10 inhibits DNA-binding activity of Rv3160c, a putative TetR-like transcription factor, and results in increased expression of Rv3161c, a protein of unknown function related to oxygenases found in other bacteria. When knocked out, *Δrv3161c* mutants became more tolerant to both INH and lysozyme, indicating that the Rv3161c plays an active role in both INH potentiation and maintenance of cell wall integrity. However, the *Δrv3161c* mutants can still be sensitized to both INH and lysozyme by C10, excluding the potential involvement of *rv3160c-rv3161c* in the C10 effect.

## Results

### Expression of the *rv3160c-rv3161c* operon is upregulated in response to C10

In our previous study, the small molecule C10 was discovered to reduce tolerance to oxidative stress, acid stress and to the first line antibiotic INH in *Mtb*^6^. Furthermore, C10 could restore INH sensitivity in otherwise INH-resistant *katG* mutants^6^. C10 also perturbed respiratory homeostasis, but its mechanism of action is unclear^6^.

In order to identify the target(s) of C10 and discover the mechanism behind its INH potentiation, we re-visited our previous RNA-seq data and looked closer at the top genes induced by C10. The *rv3160c* and *rv3161c* genes, positioned within a proposed two-gene operon^9,10^, were among the top 5 genes upregulated by C10 under hypoxic growth conditions (13-fold and 12-fold, respectively)^6^. These genes particularly caught our attention, as they have been shown to be induced by triclosan, an antibacterial that inhibits InhA, resulting in deficient mycolic acid synthesis in *Mtb*, similar to INH^11,12^.

**Figure 1:**
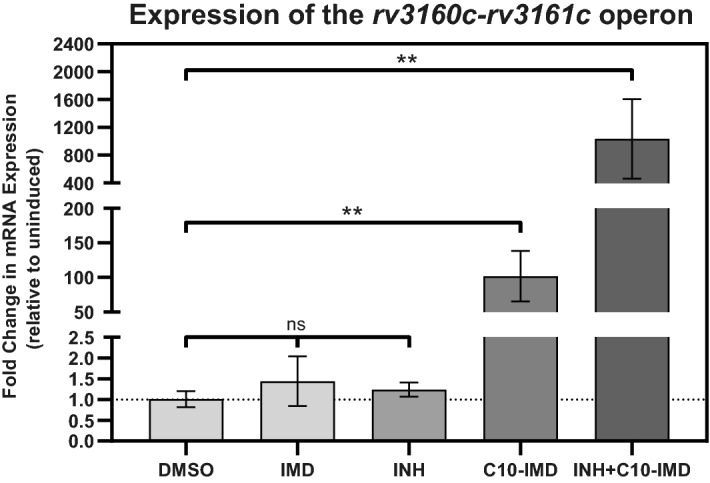
Expression of the *rv3160c-rv3161c* operon is significantly induced by C10-IMD. *Mtb* Erdman WT was cultivated in Sauton’s medium at 37 °C until its OD_600_ reached 0.3. The cells were then exposed to 0.1% DMSO, 0.1 µg/ml INH and 25 µM C10-IMD separately or in combination for 48 h at 37 °C. Differences in expression of the *rv3160c-rv3161c* operon were determined by qRT-PCR analysis performed by targeting *rv3161c* coding region. Expression of the *sigA* gene was determined by qRT-PCR analysis and used as housekeeping control. Relative fold-change in *rv3160c-rv3161c* operon expression was calculated for each biological replicate by the Livak (2^−ΔΔCt^) method. Bar graphs were plotted based on average and standard deviation obtained from three independent biological replicates and statistical analysis comparing to the DMSO control was determined by one-tail *t*-test that was performed using GraphPad Prism version 8.4.3 for Windows, GraphPad Software, San Diego, California USA, www.graphpad.com (**p < 0.01).

According to the RNA-seq results, upregulation of the *rv3160c-rv3161c* operon by C10 was observed under hypoxic growth conditions but not under aerated conditions. To investigate this further, we performed quantitative real-time PCR (qRT-PCR) analysis of the *rv3160c-rv3161c* operon in response to C10 molecule under aerobic conditions. To improve solubility of C10, we used a C10-imidazole salt (C10-IMD) instead of C10 during this study (Fig. S1)^6^. qRT-PCR data showed that expression of the *rv3160c-rv3161c* operon increases 102 ± 32-fold under aerated growth conditions upon exposure to 25 µM C10-IMD for 48 h (Fig. 1). Furthermore, expression of this operon was induced even further, 1035 ± 571-fold upon exposure to a combination of 25 µM C10-IMD and 0.1 µg/ml INH for 48 h (Fig. 1). There were no significant changes in expression of the operon upon exposure to either 0.1 µg/ml INH or 25 µM IMD alone for 48 h (Fig. 1). These findings suggest that INH and C10-IMD have a synergistic role in the induction of the *rv3160c-rv3161c* operon. Based on this, we decided to investigate the *rv3160c-rv3161c* operon further.

### Rv3160c is a TetR-like transcription repressor of the *rv3160c-rv3161*c operon

The *rv3160c* gene encodes a putative TetR family transcriptional regulator (TFTR) and is uniquely found in mycobacteria causing TB^13^. Mycobacterial genomes are highly enriched with several TFTRs, of which the majority act as repressors of gene expression^13^. Even though Rv3160c has been suggested to be a TFTR based on sequence domain similarities to other TFTRs, its function and possible target(s) have not been described. The majority of the TetR-like transcriptional repressors are known to regulate their neighboring genes and often to be co-transcribed with their regulatory targets^13^. Several previous studies have suggested that the *rv3160c* and the *rv3161c* genes are located within the same operon based on their correlated gene expression in response to various environmental factors^11,14–19^. However, a genome wide study suggested the *rv3160c* gene to have its own independent transcriptional start site (TSS) starting at 406 bp upstream of the *rv3160c* open reading frame (ORF), overlapping with the *rv3161c* ORF^20^. To determine which promoter region was responsible for the transcription of *rv3160c* in our conditions, we measured the expression levels of the *rv3160c* gene while targeting the region between the *rv3160c-rv3161c* operon TSS and the recently proposed independent TSS of the *rv3160c* gene with a CRISPRi construct (Fig. S2). We found that the expression of the *rv3160c* gene was significantly reduced suggesting that *rv3160c* is mainly co-transcribed with the *rv3161c* gene (Fig. S2). However, the existence of additional TSSs, perhaps of importance in response to other conditions, cannot be excluded based on this data.

**Figure 2:**
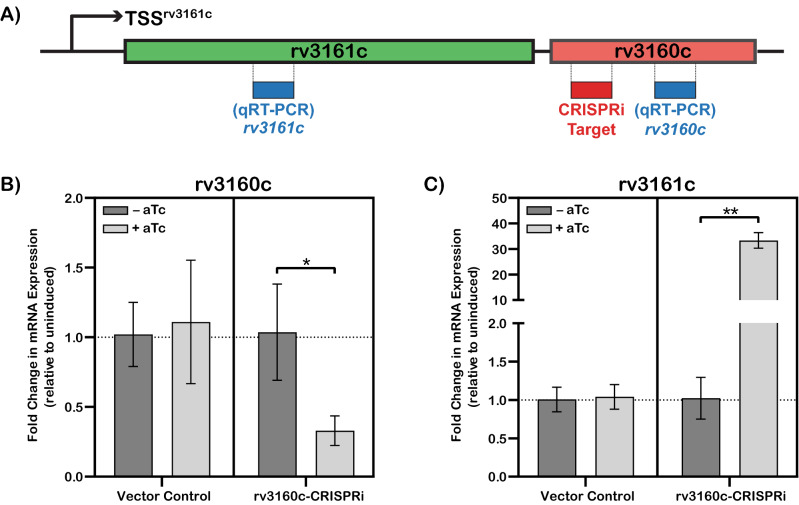
Rv3160c acts as transcriptional repressor of the *rv3160c-rv3161c* operon. (**A**) Schematic representation of the *rv3160c-rv3161c* operon and the relative positions of the targets for CRISPRi and qRT-PCR. (**B**,**C**) qRT-PCR analysis of *rv3160c* and *rv3161c* mRNA expression in *Mtb* Erdman WT strain harboring either vector control (pJR965) or the *rv3160c-CRISPRi* (pSA253) construct in absence (−) or presence (+) of 100 ng/ml aTc. *sigA* mRNA expression was used as control and relative fold change in *rv3160c* and *rv3161c* mRNA expression were calculated for each biological replicate by the Livak (2^−ΔΔCt^) method. Average value of 3 technical replicates was used for each biological replicate. Bar graphs were plotted based on average and standard deviation obtained from three independent biological replicates and statistical analysis was determined by one-tail *t*-test that was performed using GraphPad Prism version 8.4.3 for Windows, GraphPad Software, San Diego, California USA, www.graphpad.com (*p < 0.05, **p < 0.01).

We designed an experiment to test whether expression of the neighboring *rv3161c* gene was regulated by Rv3160c (Fig. 2A). We reduced expression of *rv3160c* gene (~ threefold) by *rv3160c*-CRISPRi (Fig. 2B), which resulted in a 33 ± 3-fold increased expression of the *rv3161c* gene (Fig. 2C). This data shows that decreasing *rv3160c* expression results in an increase in *rv3161c* expression, although it does not show whether this occurs through direct regulation or not.

**Figure 3:**
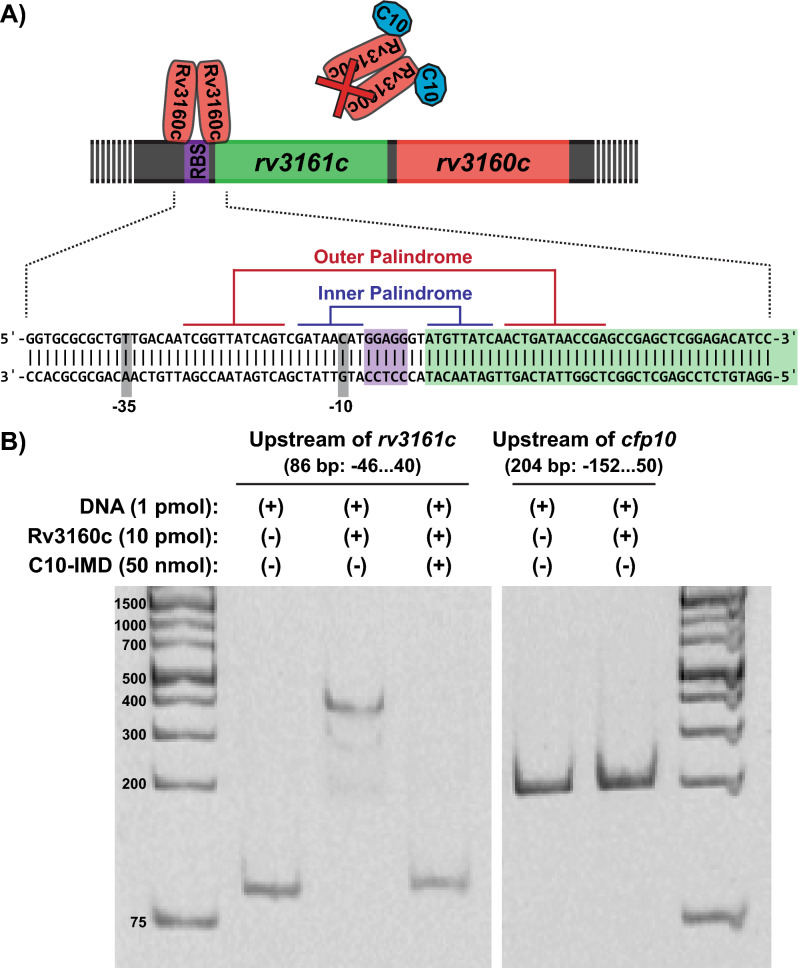
Binding of Rv3160c to the *rv3160c-rv3161c* operon is blocked by C10-IMD. (**A**) Schematic depicting model of the Rv3160c dimer binding to the palindromic sequence present in the *rv3161c* upstream fragment. (**B**) EMSA assay using an 86 bp *rv3161c* upstream fragment (1 pmol; lanes 1–3) and a 204 bp *cfp10* upstream fragment as negative control fragment (1 pmol; lanes 4–5). Lane 1 and 5: no protein added. Lanes 2, 3 and 5: binding reaction with 10 pmol Rv3160c. Lane 3: 50 nmol C10-IMD. DNA Ladder: Thermo Scientific GeneRuler 1 kb Plus (SM1331).

TFTRs typically bind to palindromic motifs as dimers, thus we searched for such motifs in the upstream sequence of the *rv3160c-rv3161c* operon. We identified a stretch of 50 bp containing 12-bp and 8-bp inverted repeats starting at the − 29 bp upstream of the operon (Fig. 3A). We confirmed that Rv3160c binds to a 142 bp DNA fragment containing these palindromic motifs, resulting in a DNA shift using the electrophoretic mobility shift assay (EMSA) (Fig. S3). We then digested the 142 bp DNA fragment with different restriction enzymes to narrow down the region that is targeted by Rv3160c (Fig. S3). We found that that presence of both inverted repeats as well as the − 10 region were necessary for an efficient interaction, thus we decided to use 86 bp DNA fragment containing both these palindromic motifs for the remaining EMSAs (Fig. 3B). The binding of Rv3160c to these palindromes would block access of the RNAP to the − 10 sigma factor binding site and repress transcription of the *rv3161c*. To test if the palindromic motifs were important for Rv3160c binding, we performed EMSAs with modified DNA fragments. We used a higher concentration of Rv3160c (100 pmol) in these assays compared to those presented in Fig. 3 (10 pmol) to ensure the protein was in excess to detect lower affinity binding events. In these experiments, we observed that our DNA dye bound to purified Rv3160c (Fig. S4), obscuring the shifted DNA band. Nevertheless, this interaction would not interfere with the migration of the band in the acrylamide gels as staining took place after the gel runs were completed. We evaluated Rv3160c binding by quantifying the amount of unbound DNA in the presence of Rv3160c as a percentage of the unbound DNA in the absence of Rv3160c for each DNA construct. To test if the inner palindrome is important for Rv3160c binding, we shuffled the 8 bp inverted repeats in the inner palindrome, without eliminating the palindrome structure or base composition (Fig. S5A). Rv3160c showed decreased binding to the fragment harboring a shuffled inner palindrome, leaving 83% of the DNA unbound, as compared to the unmodified fragment, where only 8% of the DNA remains unbound by Rv3160c (Fig. S5B). We then incubated Rv3160c with DNA fragments harboring a shuffled outer palindrome or harboring both shuffled inner and outer palindromes and found that nearly all of the DNA remained unbound in the presence of Rv3160c (Fig. S5). Furthermore, by modifying the two non-matching nucleotides located between the inner and outer palindromes to create a single 21 bp inverted repeat, we found that Rv3160c was still able to bind most of the DNA fragment, leaving 37% of the DNA unbound (Fig. S5A). These findings demonstrate that the ability of Rv3160c to bind the sequence upstream of *rv3161c* is dependent on the sequence of both inverted repeats.

### C10-IMD inhibits the interaction between Rv3160c and the *rv3161c* upstream target sequence

**Figure 4:**
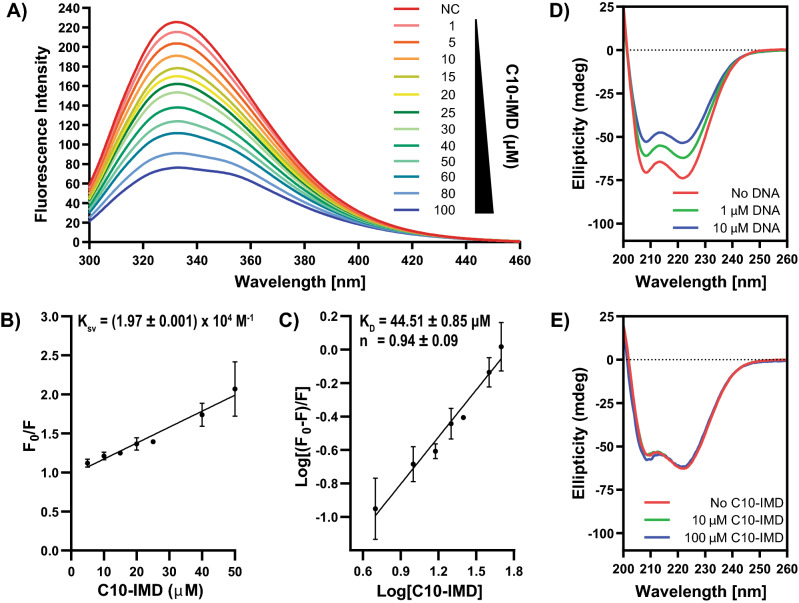
C10-IMD binds to a single site in Rv3160c and the interaction does not cause changes in the secondary structure of the protein. (**A**) Fluorescence quenching spectra of Rv3160c. The 10 µM Rv3160c in PBS solution is excited and quenching of fluorescence is recorded in the presence of varying concentrations of C10-IMD (1–100 μM). (**B**) Stern–Volmer plot of decrease in mean fluorescence intensity of Rv3160c in the presence of varying concentrations of C10-IMD derived from three independent experiments. The dynamic quenching rate constant K_sv_ is evaluated from the slope of the line. (**C**) Logarithmic plot of mean relative fluorescence quenching of Rv3160c derived from three independent experiments against logarithmic concentrations of C10-IMD. K_D_ is calculated from the intersection of the line with the y axis and the number of binding sites (n) from the slope of the line. (**D**) CD spectra of Rv3160c in the presence of 1 µM or 10 µM 86 bp *rv3161c* upstream fragment (DNA) (Fig. 2C). (**E**) CD spectra of Rv3160c in the presence of 10 µM or 100 µM C10-IMD. Error bars in (**B**) and (**C**) indicate standard deviation.

As mentioned above, expression of the *rv3160c-rv3161c* operon is induced by C10-IMD. Thus, we hypothesized that C10-IMD might interfere with Rv3160c function. Indeed, we found that Rv3160c was no longer capable of interacting with the sequence upstream of *rv3161c* in the presence of C10-IMD in the EMSA (Fig. 3B). To investigate whether C10-IMD interacted directly with Rv3160c, we analyzed the effect of C10-IMD on Rv3160c tryptophan fluorescence emission. There are 3 tryptophan residues available in Rv3160c and the binding interaction between C10-IMD and Rv3160c was studied using tryptophan fluorescence. The presumption is that if C10-IMD binds Rv3160c and some of the tryptophan residues are in close proximity to the C10-IMD binding site, compound binding might affect the intrinsic fluorescence of Rv3160c. Rv3160c was titrated with increasing concentrations of C10-IMD and relative fluorescence intensity was found to decrease in a concentration-dependent manner (Fig. 4A). The fluorescence quenching was further analyzed by Stern–Volmer equation (Eq. 1, see “Methods” section) to determine the Stern–Volmer quenching constant (K_sv_) (Fig. 4B), which signifies the equilibrium binding constant for complex formation in the light-induced electronic excited state resulting in the quenching of intrinsic fluorescence of tryptophan. In this study, the Stern–Volmer plot analyzes the type of fluorescence quenching of tryptophan residues in Rv3160c by plotting relative emission intensity against C10-IMD concentration. In the case of Rv3160c, a tryptophan might be buried in the protein, whose fluorescence quenching depends on the microenvironment, solvent accessibility, and binding of C10-IMD. Overall, the tryptophan fluorescence quenching is a combined contribution of the microenvironment of all tryptophan residues in the presence of C10-IMD. If tryptophan residues are in close proximity of C10-IMD binding site, C10-IMD binding leads to fluorescence quenching of tryptophan while the fluorescence of other tryptophan residues that are not involved in C10-IMD binding would not be quenched. The Stern–Volmer plot, therefore, helps to understand the binding process leading to quenching phenomena^21^. To calculate the binding dissociation constant (K_D_) and the number of binding site (n), the logarithmic Eq. (2) (see “Methods” section) was used. The log(K_b_) (K_b_ denotes binding association constant) value had been determined from the intercept of the plot, shown in Fig. 4C. The binding dissociation constant (K_D_) was found to be 44.51 ± 0.85 µM using Eq. (3). The number of binding sites for the C10-IMD in Rv3160c was found to be 0.94 ± 0.09 suggesting one binding site of C10-IMD in Rv3160c.

TFTRs usually consist of two domains: one signal-receiving and the other one DNA-binding. Interactions of TFTRs with their target DNAs often lead to structural changes, especially in the DNA-binding domain^22–25^. Similarly, specific molecules can bind to the regulatory domain of TFTRs, resulting in conformational changes that make TFTRs incapable of binding their DNA targets^26,27^. According to our EMSA experiments, Rv3160c cannot bind to its DNA target upon addition of C10-IMD. Thus, we investigated whether interaction with C10-IMD results in conformational changes in Rv3160c using circular dichroism (CD) spectroscopy. We discovered that the ellipticity of Rv3160c was reduced upon addition of its DNA target, indicating changes in the secondary structure (Fig. 4D). In contrast, no change in the ellipticity of Rv3160c was observed in the presence of C10-IMD (Fig. 4E), indicating that C10-IMD did not alter the secondary structure of Rv3160c in the absence of DNA. There is a slight change in the ellipticity in the presence of C10-IMD approximately at 208 nm in Fig. 4E does not have much impact on overall secondary structure of Rv3160c.

### Deletion of *rv3161c* results in increased sensitivity to INH and lysozyme

**Figure 5:**
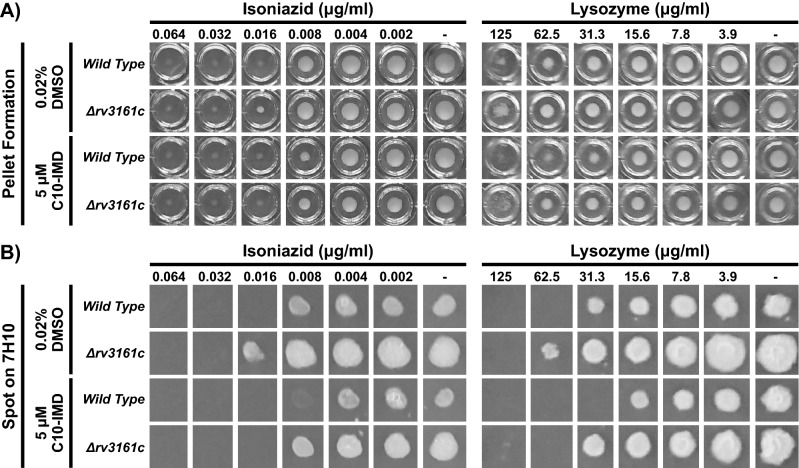
Deletion of the *rv3161c* gene results in higher resistance to isoniazid and lysozyme. (**A**) Representative images showing effect of INH and lysozyme on bacterial growth scored by pellet formation. *Mtb* Erdman WT and *∆rv3161c* strains were incubated 2 weeks in Sauton’s medium with 0.02% DMSO or 5 µM C10-IMD in addition to various concentration of INH (left; 0.002–0.064 µg/ml) or lysozyme (right; 3.9–125 µg/ml). (**B**) Representative images showing effect of INH and lysozyme treatment on bacterial survival scored by spotting bacteria exposed to INH (left; 0.002–0.064 µg/ml) or lysozyme (right; 3.9–125 µg/ml) in the presence of 0.02% DMSO or 5 µM C10-IMD on antibiotic-free 7H10 plates.

C10-IMD directly binds Rv3160c, a TetR-like transcriptional repressor, which could be leading to the increased expression of the *rv3160c-rv3161c* operon in the presence of C10-IMD. We hypothesized that INH potentiation by C10-IMD might be regulated through the cellular concentration of Rv3161c, a putative oxygenase. To test this, we used recombineering to generate a *rv3161c* knock-out strain and tested its sensitivity to INH. Both wild type and *Δrv3161c* strains were incubated for 2 weeks at 37 °C with varying concentrations of INH in U-bottomed 96-well plates. After 2-weeks incubation, we scored the pellet formations in each well as a semi-quantitative indication of bacterial growth and spotted them on 7H10 plates followed by additional 3-weeks of incubation at 37 °C to assess the presence of viable bacteria. We discovered that the *Δrv3161c* strain was more resistant to INH in comparison to wild type, as it could survive up to double the INH concentration (0.016 µg/ml) (Fig. 5). We also observed that addition of 5 µM C10-IMD potentiated INH sensitivity in both wild type and *Δrv3161c* strains, demonstrating that the effect of C10-IMD on INH potentiation occurs independently of Rv3161c (Fig. 5).

Previous studies show that expression of the *rv3160c*-*rv3161c* operon is upregulated in the lipid-rich environments^18^ and upon treatment with cell wall synthesis inhibitors^11,17,19^, raising the question of whether these genes play a role in cell wall integrity. We set up a similar growth and survival assay to test the cell wall integrity of both wild type and *Δrv3161c* strains by incubating them in the presence of varying concentrations of lysozyme, an antimicrobial enzyme that cleaves the peptidoglycan component of the bacterial cell wall. The *Δrv3161c* strain survived exposure up to double the lysozyme concentration (62.5 µg/ml) compared to the wild type strain (Fig. 5). In addition, we found that both the wild type and *Δrv3161c* strains were more sensitive to lysozyme in the presence of 5 µM C10-IMD (Fig. 5).

## Discussion

Expression of the *rv3160c-rv3161c* operon has been reported to be induced by various anti-microbial agents such as triclosan^11,19^, thioridazine^16,17,19^, SRI#967^15^, SRI#9190^15^ and chlorpromazine^19^. Rv3161c encodes a putative oxygenase that shares high amino acid sequence identity (32–62%) with other Rieske oxygenases found in several organisms. Rieske oxygenases are known to be involved in the degradation of arenes, therefore it has been suggested that Rv3161c might hydroxylate the benzene ring structures found in these anti-microbial agents resulting in detoxification^11,15,17^. However, neither deletion nor overexpression of the *rv3161c* gene influenced the minimal inhibitory concentration (MIC) of triclosan or other anti-microbial agents^28^. Therefore, upregulation of *rv3161c* expression was suggested to be a failed attempt of the bacteria to counteract these drugs^28^. In another study, mutations in the *rv3161c* gene were found to cause resistance against the 2,4-diaminoquinazoline (DAQ) anti-tubercular agent and Rv3161c was proposed to convert pro-drug DAQ into its active form^29^. Despite all of these previous studies, the mechanism of action of the Rv3161c in response to antimicrobial agents is still unclear.

We discovered that *rv3161c* is highly expressed in response to the C10-IMD compound that previously has been shown to potentiate INH sensitivity^6^. Induced *rv3161c* expression was also observed as a response to 2-aminoimidazoles (2-AI), a class of molecules that potentiate sensitivity to β-lactam antibiotics by increasing cell permeability^14^. Several studies have suggested that Rv3161c is involved in lipid metabolism and cell wall biogenesis^11,17–19^. In this study, we found that *Δrv3161c* mutants become more resistant to INH as well as lysozyme, an enzyme degrading the peptidoglycan layer of the cell wall (Fig. 5). We hypothesize that elevated Rv3161c may disturb the cell wall integrity and increase cell permeability, resulting in increased INH and lysozyme sensitivity. The *Δrv3161c* mutants can still be sensitized against both INH and lysozyme by C10-IMD, demonstrating that C10-IMD functions independent of Rv3161c to elicit these effects (Fig. 5). We tried to generate a *Δrv3160c* mutant to test whether elevated Rv3161c in the absence of C10-IMD could increase sensitivity against INH and lysozyme, however we failed to generate such a mutant in several attempts.

In this study, we clarified the function of Rv3160c as a TetR-like transcriptional repressor that regulates *rv3161c* expression. Despite the fact that several publications have suggested the *rv3160c-rv3161c* operon to be involved in key processes such as drug detoxification, lipid metabolism and cell wall biogenesis^11,14–19,29^, the function of the Rv3161c remains unknown. Further investigation aiming to elucidate the function of Rv3161c will lead to important insights into *Mtb* biology.

## Methods

### Bacterial strains and growth conditions

WT *E. coli* DH5α was grown in Luria Broth (Difco) as shaking cultures at 37 °C and kanamycin at 50 µg/ml was used for selection when required.

WT *Mtb* Erdman and the *rv3161c* knockout with *Mtb* Erdman background were grown in either Middlebrook 7H9 medium (BD Difco) supplemented with 0.05–0.1% Tween80 and 10% ADS (0.5% albumin, 0.2% dextrose and 0.085% saline) or Sauton’s medium [0.0005% (w/v) KH_2_PO_4_, 0.0005% (w/v) MgSO_4_, 0.004% (w/v) l-asparagine, 0.06% glycerol, 0.00005% (w/v) ferric ammonium citrate, 0.002% (w/v) citric acid, 0.0001% ZnSO_4_, pH 7] supplemented with 0.1% Tween80 as liquid shaking cultures at 37 °C. These strains were also grown on Middlebrook 7H10 solid medium (Remel) supplemented with 0.05% Tween80, 0.2–0.5% Glycerol and 10% ADS at 37 °C. Kanamycin at 20 µg/ml and anhydrotetracycline (aTc) at 100 ng/ml were used when required. All the strains used in this work are listed in Table S1.

### Construction of *rv3160c-*CRISPRi and *rv3161c*-CRISPRi strains

To investigate regulation of the *rv3160c-rv3161c* operon, we silenced expression of the *rv3160c* and the *rv3161c* genes individually by using the dCas9_Sth1_ CRISPRi system^30^. This system utilizes a catalytically inactivated CRISPR1 *cas9* allele and provides an RNA-guided platform for sequence-specific control of gene expression. The single guide RNAs (sgRNAs) for each gene were determined and ranked by PAM score. Forward and reverse oligonucleotides (Table S2) corresponding to the top-ranked sgRNA sequence for each gene were annealed together to form double stranded DNA fragments and subsequently cloned into the BsmBI site of pLJR965 as described before^30^. The resulting plasmids, named pSA253 (targeting *rv3160c*) and pSA256 (targeting *rv3161c*), were electroporated into *Mtb* Erdman to obtain TB3 and TB4 respectively.

### RNA isolation and cDNA synthesis

*Mtb* Erdman strains harboring pLRT965 (empty vector), pSA253 (targeting *rv3160c*) or pSA256 (targeting *rv3161c*) were grown in 7H9 medium supplemented with 0.05% Tween80, 10% ADS and 20 µg/ml kanamycin until their optical density (OD_600_) reaches to 0.3 in the presence of aTc (100 ng/ml) to induce expression of gene-specific sgRNA. Same strains were also cultivated similarly but in the absence of aTc as non-induced controls. Equivalents of 6 OD_600_-unit cells were harvested by centrifugation and resuspended in 1 ml TRI Reagent (Invitrogen). Cell suspensions were transferred into screw-cap homogenizing tubes containing 0.4 ml of 0.1 mm Zirconia/Silica beads (BioSpec) and lysed in a bead beater 8 times for 30 s with a 1-min rest on ice between each cycle. After a centrifugation at 13,000 g for 45 s, supernatants were transferred into new tubes and mixed with 300 µl chloroform followed by incubation at room temperature for 2 min. The samples were centrifuged at 13,000 g for 5 min and aqueous phase were transferred into new tubes. An equal volume of absolute ethanol was mixed with each sample and subsequently transferred into RNeasy columns (Qiagen) followed by purification according to the manufacturer’s instruction. Isolated RNA samples were then DNase-treated with TURBO DNA-free kit (Invitrogen) to eliminate any genomic DNA contamination. cDNA was prepared using qScript cDNA Synthesis kit (Quantabio) according to the manufacturer’s instruction.

### Quantitative real-time PCR

Expression levels of *rv3160c* and *rv3161c* genes were determined by qRT-PCR with cDNA equivalent to 50 ng RNA using Maxima SYBR Green/ROX qPCR Master Mix (Thermo Scientific) and the oligonucleotides listed in Table S3. RNA samples that were not reverse transcribed were used to confirm that there were no DNA contamination, and the integrity of the amplicons were confirmed by melting curves for each sample. All qRT-PCR experiments were performed with three technical replicates derived from three biological replicates. Signals were normalized to the housekeeping *sigA* mRNA and quantified by the Livak (2^−ΔΔCt^) method^31^. The Ct values of induced samples were compared to the corresponding Ct values of uninduced samples. Statistical analysis was performed using the one-tailed *t*-test and the results were plotted as bar graphs based on average and standard deviation obtained from three biological replicates by GraphPad Prism version 8.4.3 for Windows, GraphPad Software, San Diego, California USA, www.graphpad.com.

### Electrophoretic mobility shift assay

EMSA was performed with 142 bp *rv3161c* upstream fragment (undigested or digested with BglI, HincII or TaqI), 86 bp *rv3161c* upstream fragment (wild type or altered) or 204 bp *cfp10* upstream fragment. The 142 bp *rv3161c* upstream sequence was directly amplified from chromosomal DNA with BS3161F and BS3161R oligonucleotides. Complementary oligonucleotides bearing the 86 bp *rv3161c* upstream sequence (wild type or altered) were annealed and amplified with UniPal_F and UniPal_R oligonucleotides (Table S4). *cfp10* upstream sequence was also directly amplified from chromosomal DNA with EMSA_CFP10-F and EMSA_CFP10-R oligonucleotides (Table S4). The binding reactions were prepared in 10 mM Tris–HCl (pH 8.0), 150 mM KCl, 0.5 mM EDTA (pH 8.0), 0.1% Triton-X 100, 12.5% glycerol, 0.2 mM DTT, 0.1 mg/ml BSA and 10 ng/ul Poly-dIdC in the presence of 1 or 4 pmol DNA targets, 10 or 56 pmol Rv3160c (produced by the Protein Expertise Platform at Umeå University in 1× PBS) and/or 50 nmol C10-IMD and incubated for 30 min at 37 °C. Reaction mixes were then loaded onto 6% polyacrylamide gels and run at 80 V in 0.5% TBE buffer for 75 min. The gels were then stained in 3× GelRed (Biotium) buffer for 15 min followed by washing with MQ water for 15 min twice. The DNA was visualized with UV-transilluminator and signals were analyzed by Image J Software version 1.52a^32^.

### Fluorescence spectroscopy

The interaction between C10-IMD and Rv3160c was analyzed by concentration-dependent response of C10-IMD on Rv3160c tryptophan fluorescence emission measured in Hitachi F-4500 fluorescence spectrophotometer. Rv3160c emission spectra were acquired using 10 μM of protein dissolved in 1× PBS (pH 6.8) with or without adding C10-IMD and was incubated for 1 min at 37 °C in a quartz cuvette. The resulting solution was excited at 290 nm for tryptophan residue and the intrinsic fluorescence emission was scanned from 300 to 460 nm. The data from each read was then combined and plotted using the Spectragryph Software version 1.2.13^33^. The fluorescence quenching data have been corrected for inner filter effects. Fluorescence quenching was determined by analyzing the data by the classical Stern–Volmer equation:

1 $$ {\text{F}}_{0} /{\text{F }} = { 1 } + k_{{\text{q}}} \tau_{0} \left[ {\text{Q}} \right] \, = { 1} + K_{{{\text{sv}}}} \left[ {\text{Q}} \right] $$

where F_o_ and F are the fluorescence intensities in the absence and presence of the quencher, respectively. K_q_ is the bimolecular quenching rate constant and K_sv_ is the dynamic quenching constant. Average lifetime of the protein in absence of the quencher is expressed in terms of τ_o_ whereas [Q] is the concentration of the free quencher. When small molecules interact with several comparable sites in a protein, the equilibrium between liberated and bound molecules is expressed by the equation:

2 $$ {\text{log}}\left( {\left( {{\text{F}}_{0} - {\text{ F}}} \right)/{\text{F}}} \right) = {\text{log}}K_{{\text{b}}} + {\text{ n log}}\left[ {\text{Q}} \right] $$

3 $$ {\text{K}}_{{\text{D}}} = {1}/K_{{\text{b}}} $$

where K_b_ represents binding constant for quencher-protein interaction, n is the number of binding sites per molecule of Rv3160c, K_D_ denotes binding dissociation constant.

### Circular dichroism (CD) spectroscopy

CD spectroscopy was performed with Rv3160c as described earlier^34^. The native secondary structure of Rv3160c was compared using far-UV range CD spectra in presence of various concentrations of C10-IMD (10 µM and 100 µM) and 86 bp *rv3161c* upstream DNA bait (1 µM and 10 µM). The spectra were recorded using Jasco CD spectrometer at a range of 200 to 260 nm from 10 µM of Rv3160c in optically clear buffer under the nitrogen atmosphere at 25 °C. The buffer baseline corrected spectra were processed with an average of 5 scans and presented as units of mean residue ellipticity.

### Impact of isoniazid and lysozyme on bacterial growth and survival

The bactericidal/bacteriostatic properties of isoniazid and lysozyme were assayed at varying concentrations (0.002–0.064 µg/ml for INH or 3.9–125 µg/ml for lysozyme) in Sauton’s medium with 0.02% DMSO or 5 µM C10-IMD for both *Mtb* Erdman wild type and *Δrv3161c* strains as described in our previous study^35^.

## Supplementary Information

Supplementary information.
