# Supplementary material for: Mycobacterium tuberculosis Rv3160c is a TetR-like transcriptional repressor that regulates expression of the putative oxygenase Rv3161c
Source: Sci Rep. 2021 Jan 15;11:1523. doi: 10.1038/s41598-021-81104-y (PMC7810876; doi:10.1038/s41598-021-81104-y)
Supplement: Supplementary file 1 — Supplementary information. [file 41598_2021_81104_MOESM1_ESM.docx]

# Supplementary Material

***Mycobacterium tuberculosis* Rv3160c is a TetR-like transcriptional repressor that regulates expression of the putative oxygenase Rv3161c**

Hasan Tükenmez^1,2,3,4^*, Souvik Sarkar^1,3^, Saber Anoosheh^1,3^, Anastasiia Kruchanova^2,3^, Isabel Edström^2,3^, Gregory A. Harrison^5^, Christina L. Stallings^5^, Fredrik Almqvist^1,3^ and Christer Larsson^2,3,6^*.

^1^ Department of Chemistry, Umeå University, SE-90187, Umeå, Sweden.

^2^ Department of Molecular Biology, Umeå University, SE-90187, Umeå, Sweden.

^3^ Umeå Centre for Microbial Research, Umeå University, SE-90187, Umeå, Sweden.

^4^ Molecular Infection Medicine, Sweden (MIMS), Umeå University, SE-90187, Umeå, Sweden

^5^ Department of Molecular Microbiology, Washington University School of Medicine, St. Louis, MO 63110 USA

^6^ Current address: Sjöstigen 10, SE-91333, Holmsund, Sweden.

*Correspondence: hasan.tukenmez@umu.se and christer.larsson1975@gmail.com

**List of the supplementary material included:**

**Supplementary Tables:**

**Table S1.** List of *M. tuberculosis* strains used in this work.

**Table S2.** The sgRNA-specific oligonucleotides used in this work.

**Table S3.** The oligonucleotides used in this work for the qRT-PCR assays.

**Table S4.** Oligonucleotides used in EMSA assays.

**Supplementary Figures:**

**Figure S1.** Synthesis of C10-IMD.

**Figure S2.** Expression of the *rv3160c* gene mainly depends on the transcriptional start site located upstream of the *rv3160c-rv3161c* operon.

**Figure S3.** Narrowing down the region that is required for the Rv3160c binding.

**Figure S4.** Rv3160c can be stained by GelRed in the absence of DNA.

**Figure S5** Alterations in the palindromic sequence upstream of *rv3161c* inhibits Rv3160c binding.

**Table S1.** List of *M. tuberculosis* strains used in this work.

| ***M. tuberculosis* strains** | **Description** | **Reference** |
| --- | --- | --- |
| Erdman WT | Parental WT strain | ATCC 35801 |
| TB10 | Erdman WT with pLJR965 | This work |
| TB3 | Erdman WT with pLRT965::sgRNA-*rv3160c* (pSA253) | This work |
| TB4 | Erdman WT with pLRT965::sgRNA-*rv3161c* (pSA256) | This work |

**Table S2.** The sgRNA-specific oligonucleotides used in this work.

| **Oligonucleotides** | **Sequence (5’-3’)** | **Target** | **Plasmid** |
| --- | --- | --- | --- |
| OLG05 | GGGAGCAAGGATATCGCGTTTGGC | *rv3160c* | pSA253 |
| OLG06 | AAACGCCAAACGCGATATCCTTGC |  |  |
| OLG11 | GGGAATTCCAGGTGGCCACGGCGAACT | *rv3161c* | pSA256 |
| OLG12 | AAACAGTTCGCCGTGGCCACCTGGAAT |  |  |

**Table S3.** The oligonucleotides used in this work for the qRT-PCR assays.

| **Target Gene** | **Forward Oligonucleotides (5’-3’)** | **Reverse Oligonucleotides (5’-3’)** | **Product Size** |
| --- | --- | --- | --- |
| *rv3160c* | CTCGACGCGAGACATTG | GGCATCGCGGTGATGT | 147 bp |
| *rv3161c* | GCATGTCGACCAAGTCGC | GCAGGTCAACGTGTCGATG | 175 bp |
| *rv2703 (sigA)* | CCATCCCGAAAAGGAAGACC | TCGAGGTCTGGTTCAGCGTC | 212 bp |

**Table S4.** Oligonucleotides used in EMSA assays.

| **Oligo_ID** | **Description** | **Sequence (5’-3’)** |
| --- | --- | --- |
| BS3161F | Oligonucleotides to amplify a 142 bp *rv3161c* upstream fragment (wild type or altered) | GTTGGCTACCTACGTGCTG |
| BS3161R |  | GAGCTCGGCTCGGTTATCA |
| UniPal_F | Universal oligonucleotides to amplify an 86 bp *rv3161c* upstream fragment (wild type or altered) | GGTGCGCGCTGTTGACAA |
| UniPal_R |  | GGATGTCTCCGAGCTCGGC |
| UniPal_WT-F | Forward and reverse oligonucleotides annealed together to form an 86 bp wild type *rv3161c* upstream fragment without any alteration. | GGTGCGCGCTGTTGACAATCGGTTATCAGTCGATAACATGGAGGGTATGTTATCAACTGATAACCGAGCCGAGCTCGGAGACATCC |
| UniPal_WT-R |  | GGATGTCTCCGAGCTCGGCTCGGTTATCAGTTGATAACATACCCTCCATGTTATCGACTGATAACCGATTGTCAACAGCGCGCACC |
| UniPal_In-F | Forward and reverse oligonucleotides annealed together to form an 86 bp altered *rv3161c* upstream fragment with shuffled inner palindrome. | GGTGCGCGCTGTTGACAATCGGTTATCAGTCTAGACTAAGGAGGGTTTAGTCTAAACTGATAACCGAGCCGAGCTCGGAGACATCC |
| UniPal_In-R |  | GGATGTCTCCGAGCTCGGCTCGGTTATCAGTTTAGACTAAACCCTCCTTAGTCTAGACTGATAACCGATTGTCAACAGCGCGCACC |
| UniPal_Out-F | Forward and reverse oligonucleotides annealed together to form an 86 bp altered *rv3161c* upstream fragment with shuffled outer palindrome. | GGTGCGCGCTGTTGACAAGTGTCTGCATTACGATAACATGGAGGGTATGTTATCATAATGCAGACACGCCGAGCTCGGAGACATCC |
| UniPal_Out-R |  | GGATGTCTCCGAGCTCGGCGTGTCTGCATTATGATAACATACCCTCCATGTTATCGTAATGCAGACACTTGTCAACAGCGCGCACC |
| UniPal_Tot-F | Forward and reverse oligonucleotides annealed together to form an 86 bp altered *rv3161c* upstream fragment with single mismatched nucleotide pair alteration. | GGTGCGCGCTGTTGACAATCGGTTATCAGTGGATAACATGGAGGGTATGTTATCCACTGATAACCGAGCCGAGCTCGGAGACATCC |
| UniPal_Tot-R |  | GGATGTCTCCGAGCTCGGCTCGGTTATCAGTGGATAACATACCCTCCATGTTATCCACTGATAACCGATTGTCAACAGCGCGCACC |
| UniPal_Both-F | Forward and reverse oligonucleotides annealed together to form an 86 bp altered *rv3161c* upstream fragment with shuffled inner and outer palindrome. | GGTGCGCGCTGTTGACAAGTGTCTGCATTACTAGACTAAGGAGGGTTTAGTCTAATAATGCAGACACGCCGAGCTCGGAGACATCC |
| UniPal_Both-R |  | GGATGTCTCCGAGCTCGGCGTGTCTGCATTATTAGACTAAACCCTCCTTAGTCTAGTAATGCAGACACTTGTCAACAGCGCGCACC |
| EMSA_CFP10-F | Universal oligonucleotides to amplify a 204 bp wild type *cfp10* upstream fragment | CTCGCGCAGGAGCGTGAAGAA |
| EMSA_CFP10-R |  | TTACCTGCCTCCTGCGCGAG |


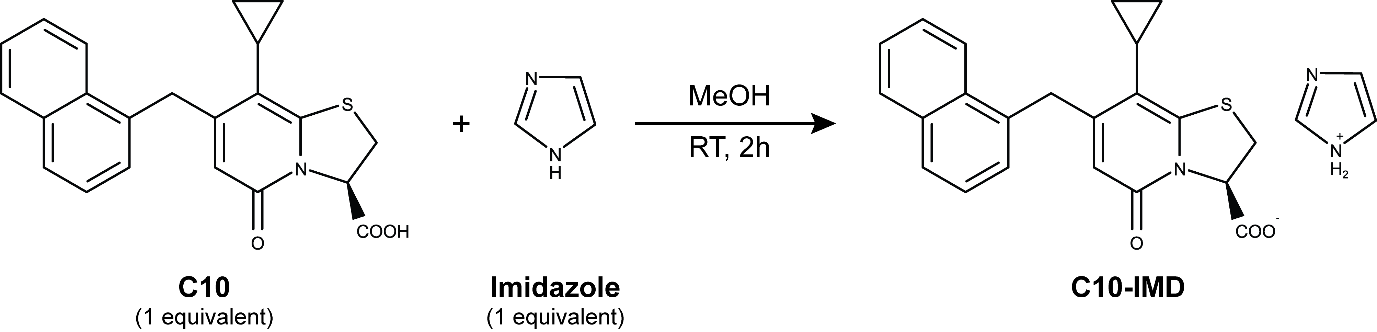


**Figure S1. Synthesis of C10-IMD.** C10 was prepared as previously described^36^. To improve its solubility, we developed a C10-imidazole salt (C10-IMD) using following method: HPLC purified C10 was dissolved in methanol at room temperature to which 1 equivalent of imidazole from a separately prepared imidazole stock solution (20 mg/ml) in methanol was added. The reaction mixture was stirred at room temperature for 2 hours in a closed round-bottomed flask. Thereafter, the solvent was evaporated under vacuum and the resulting product was dissolved in 10 ml of acetonitrile-water (1:3). The resulting mixture was subjected to freeze drying overnight to give C10-IMD as a white solid.

36 Emtenas, H., Ahlin, K., Pinkner, J. S., Hultgren, S. J. & Almqvist, F. Design and parallel solid-phase synthesis of ring-fused 2-pyridinones that target pilus biogenesis in pathogenic bacteria. *J Comb Chem* **4**, 630-639, doi:10.1021/cc020032d (2002).


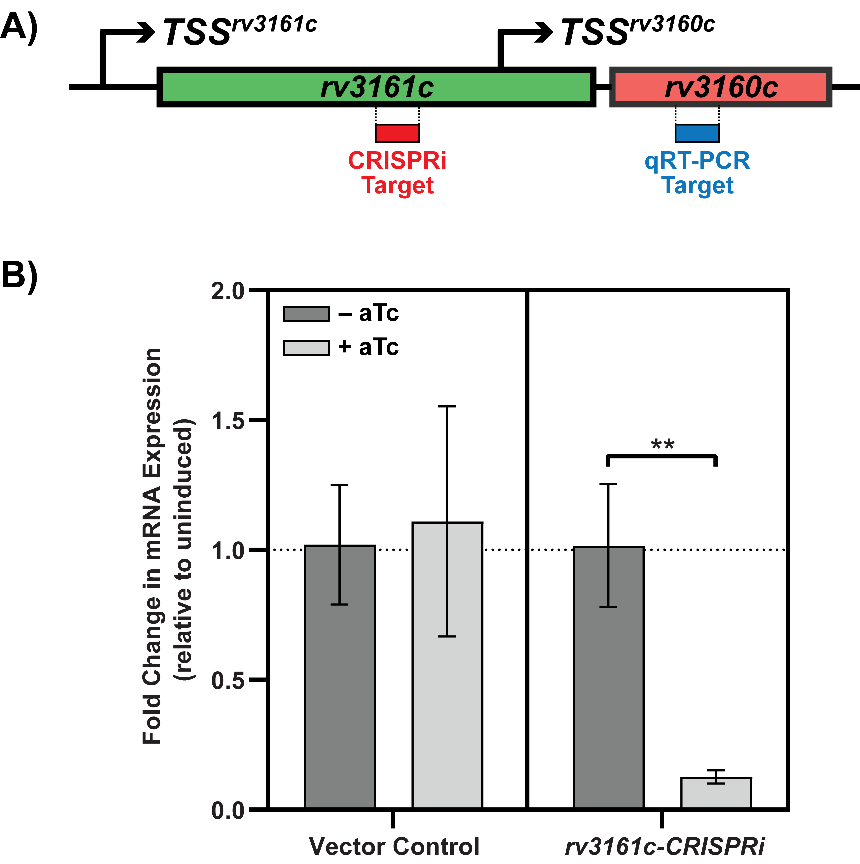


**Figure S2. Expression of the *rv3160c* gene mainly depends on the transcriptional start site located upstream of the *rv3160c-rv3161c* operon. (A)** Schematic representation of the *rv3160c-rv3161c* operon and the relative positions of the targets for CRISPRi and qRT-PCR. **(B)** qRT-PCR analysis of *rv3160c* mRNA expression in *Mtb* Erdman WT strain harboring either vector control (pJR965) or *rv3161c-CRISPRi* (pSA256) construct in the absence (-) or the presence (+) of 100 ng/ml aTc. qRT-PCR analysis of *sigA* mRNA expression was used as control and relative fold change in *rv3160c* mRNA expression was calculated for each biological replicate (as average of three technical replicates) by Livak (2^-ΔΔCt^) method. Bar graphs were plotted based on average and standard deviation obtained from three independent biological replicates and statistical analysis was determined by one-tail *t*-test that was performed using GraphPad Prism version 8.4.3 for Windows, GraphPad Software, San Diego, California USA, www.graphpad.com (** p < 0.01)**.**


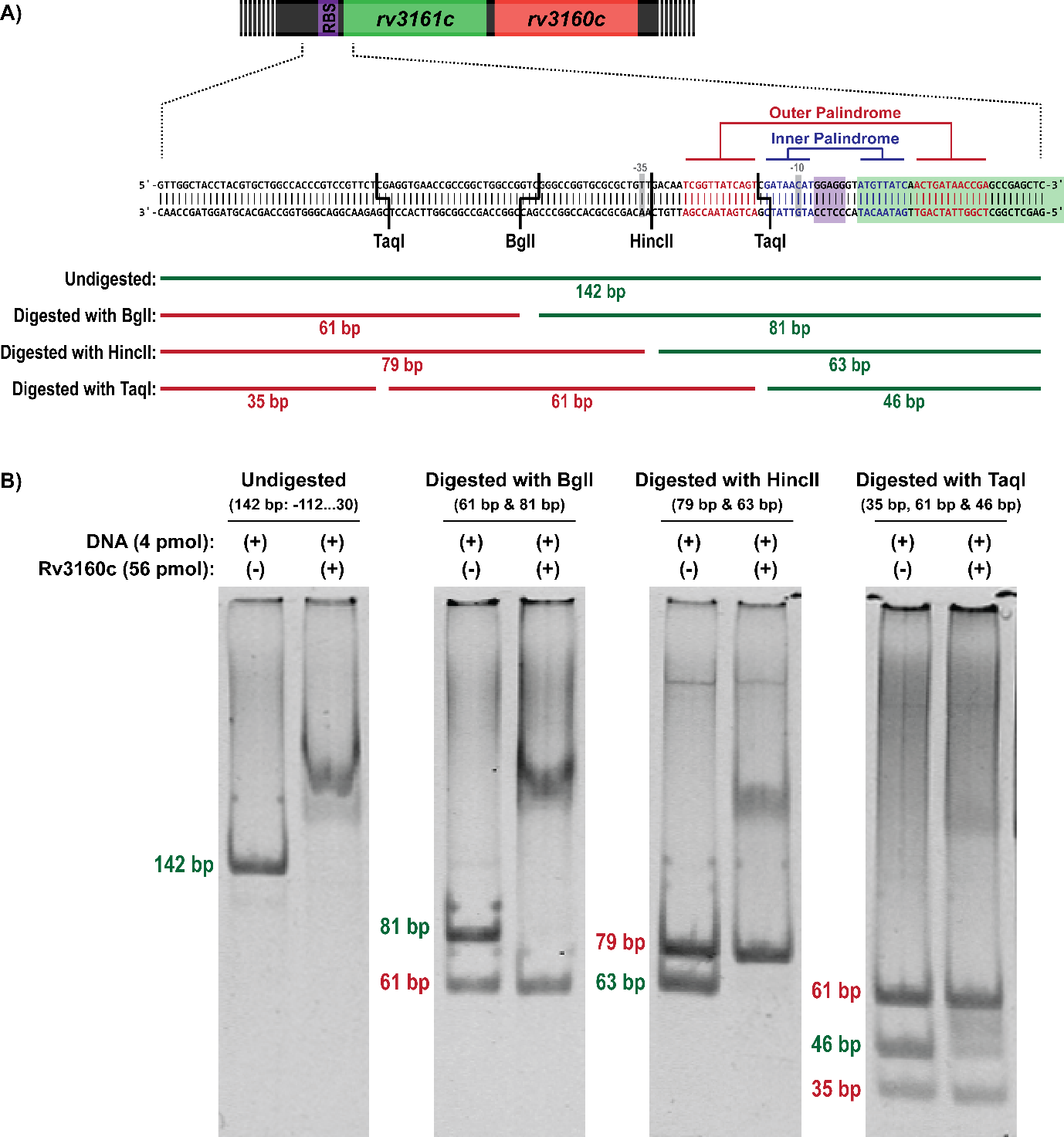


**Figure S3. Narrowing down the region that is required for the Rv3160c binding. (A)** Schematic models depicting the restriction sites present upstream of the *rv3161c* gene and the size comparison of the digested fragments. **(B)** EMSA assay using 142 bp *rv3161c* upstream fragment (4 pmol). Lane 1-2: Undigested Fragment (142 bp). Lane 3-4: BglI-digested fragments (61 bp & 81 bp). Lane 5-6: HincII-digested fragments (79 bp & 63 bp). Lane 7-8: TaqI-digested fragments (35 bp, 61 bp & 46 bp). Lane 1, 3, 5, 7: no protein was added. Lane 2, 4, 6, 8: binding reaction with 56 pmol Rv3160c. The fragments that displayed gel shift in the presence of Rv3160c are marked in green, whereas the remaining fragments are marked in red.

**
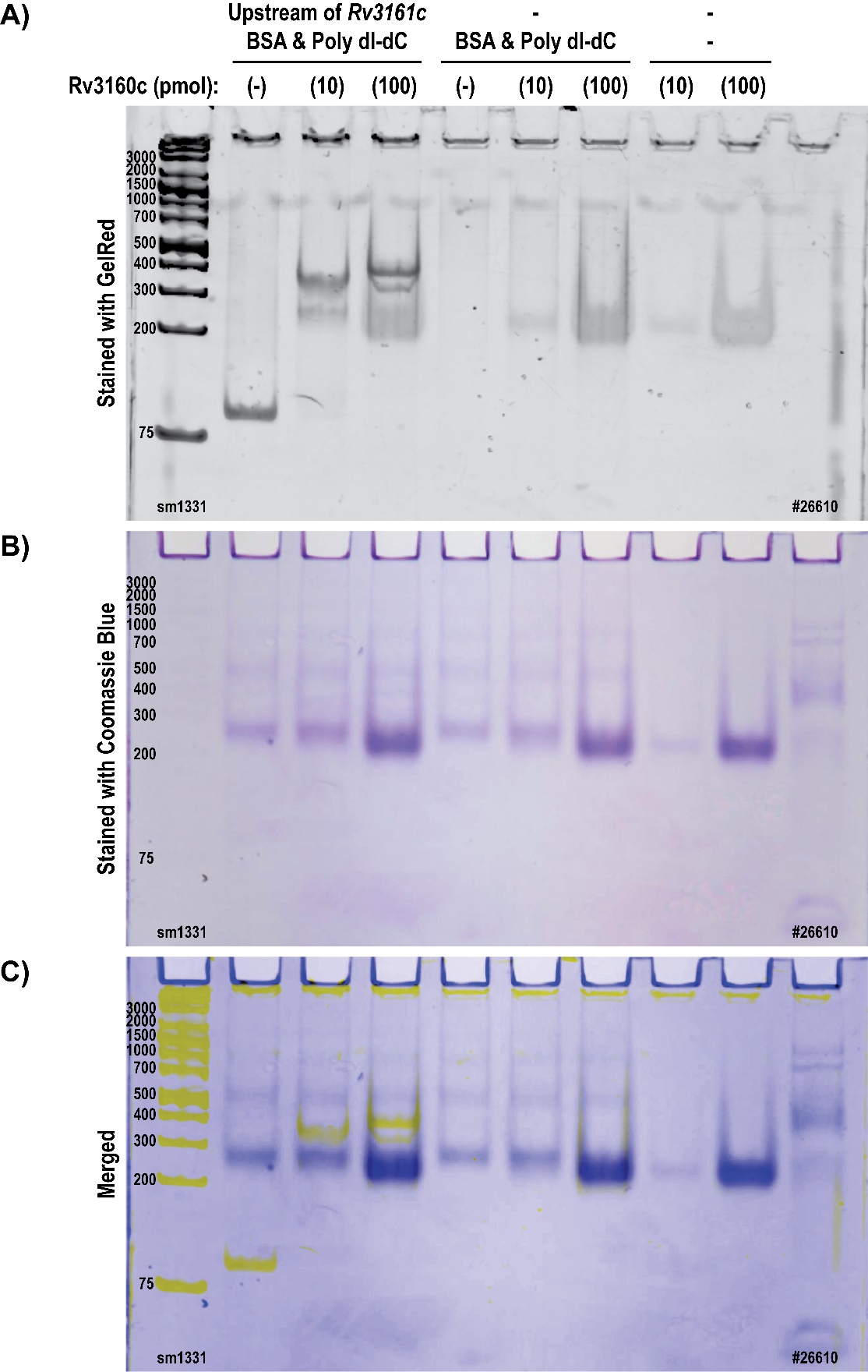
**

**Figure S4. Rv3160c can be stained by GelRed in the absence of DNA. (A)** GelRed staining of the polyacrylamide gel containing EMSA assay samples. Lane 1: Thermo Scientific GeneRuler 1 kb Plus (SM1331). Lane 2-4: the 86 bp *rv3161c* upstream fragment (1 pmol) including BSA and Poly dI-dC controls. Lane 5-7: without the 86 bp *rv3161c* upstream fragment but BSA and Poly dI-dC controls were still included. Lane 8-9: without any DNA fragment or BSA. Lane 10: Thermo Scientific Pierce Unstained Protein MW Marker (#26610). Lane 1, 2, 5, 10: no protein was added. Lane 3, 6, 8: binding reaction with 10 pmol Rv3160c. Lane 4, 7, 9: binding reaction with 100 pmol Rv3160c. **(B)** Coomassie Blue staining of the GelRed-stained polyacrylamide gel. **(C)** Merge of GelRed staining (A, Yellow Channel) and Coomassie Blue staining (B, Blue Channel) results as one.


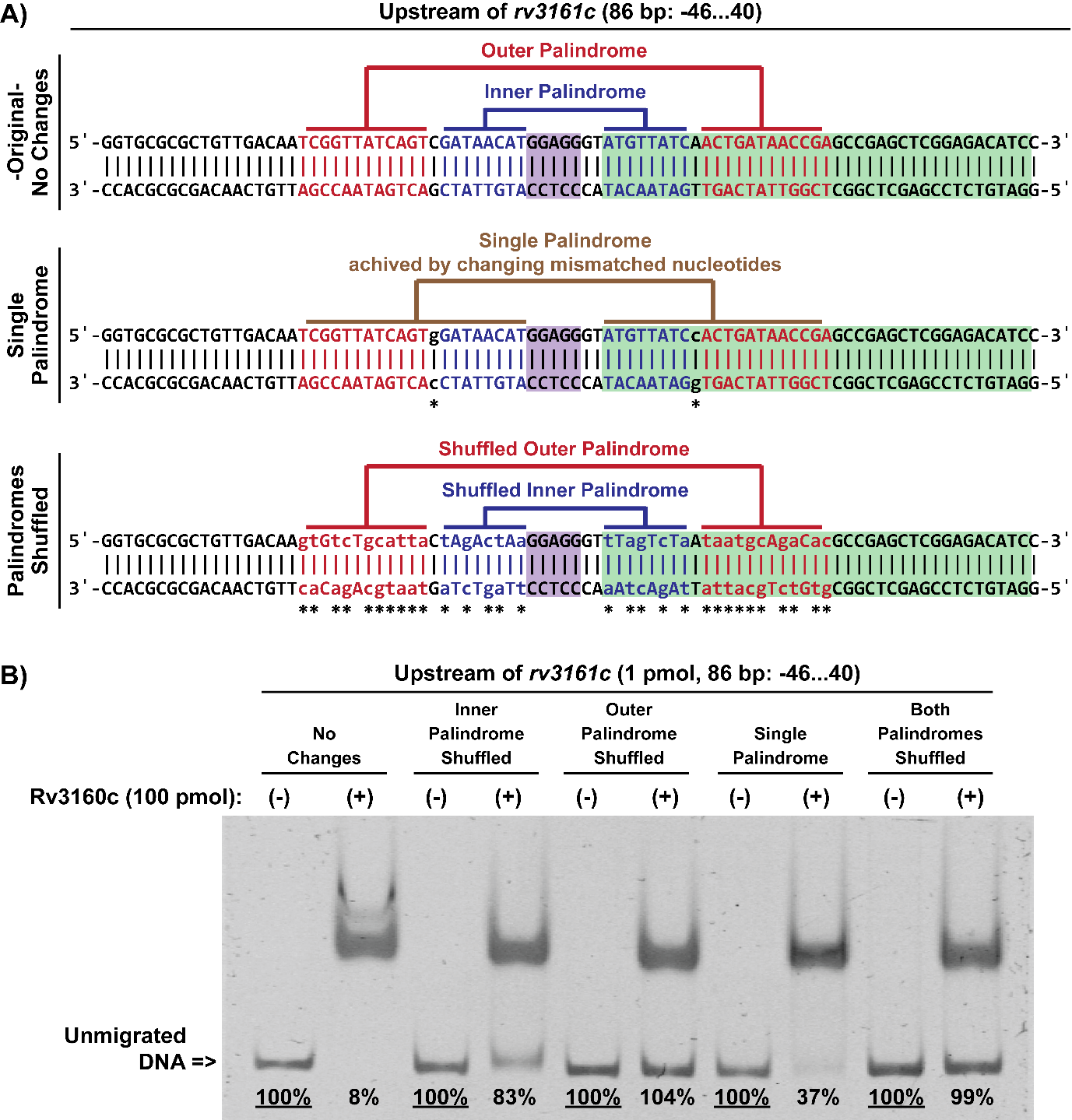


**Figure S5. Alterations in the palindromic sequence upstream of *rv3161c* inhibits Rv3160c binding. (A)** Schematic models depicting the changes done in the palindromic sequence present upstream of the *rv3161c* gene. Top: no changes, Center: single mismatching nucleotide pair located between inner and outer palindromes altered in order to achieve a perfect palindrome. Bottom: nucleotides located in the inner and outer palindromes shuffled without disturbing the palindrome or nucleotide composition. **(B)** EMSA assay using 86 bp *rv3161c* upstream fragment (1 pmol). Lane 1-2: WT fragment. Lane 3-4: Fragment with shuffled inner palindrome. Lane 5-6: Fragment with shuffled outer palindrome. Lane 7-8: Fragment with single mismatched nucleotide pair alteration (single palindrome). Lane 9-10: Fragment with shuffled inner and outer palindrome. Lane 1, 3, 5, 7, 9: no protein was added. Lane 2, 4, 6, 8, 10: binding reaction with 100 pmol Rv3160c. The unmigrated DNA bands were used for quantification as Rv3160c can also be stained by GelRed in the absence of DNA **(Figure S4)**. Signal intensity of the unmigrated DNA bands were determined by using by Image J Software version 1.52a^32^. Signal density of the unmigrated DNA band in the reaction with 100 pmol Rv3160c was expressed as a percentage of the signal density for the unmigrated DNA fragment without Rv3160c (underlined) for each DNA construct.
